# Biocompatible Interpenetrating Network Hydrogels with Dually Cross-Linked Polyol

**DOI:** 10.3390/polym17202737

**Published:** 2025-10-13

**Authors:** Ulygbek B. Tuleuov, Alexander L. Kwiatkowski, Akerke T. Kazhmuratova, Lyazzat Zh. Zhaparova, Yermauyt Nassikhatuly, Miroslav Šlouf, Andrey V. Shibaev, Viktor I. Petrenko, Senentxu Lanceros-Méndez, Yerkeblan M. Tazhbayev

**Affiliations:** 1 Faculty of Chemistry, Karaganda Buketov University, Karaganda 100028, Kazakhstan; bekalols1@gmail.com (U.B.T.); kazhmuratova@mail.ru (A.T.K.); lyazzh@mail.ru (L.Z.Z.); ermauit@gmail.com (Y.N.);; 2 Physics Department, Lomonosov Moscow State University, Moscow 119991, Russia; 3 Institute of Macromolecular Chemistry, 16200 Prague, Czech Republic; slouf@imc.cas.cz; 4 BCMaterials, Basque Center for Materials, Applications and Nanostructures, UPV/EHU Science Park, 48940 Leioa, Spain; viktor.petrenko@bcmaterials.net (V.I.P.); senentxu.lanceros@bcmaterials.net (S.L.-M.); 5 IKERBASQUE, Basque Foundation for Science, 48009 Bilbao, Spain

**Keywords:** hydrogels, interpenetrating networks, poly(vinyl alcohol), microcrystallites, tannic acid, rheology

## Abstract

Modern tissue regeneration strategies rely on soft biocompatible materials with adequate mechanical properties to support the growing tissues. Polymer hydrogels have been shown to be available for this purpose, as their mechanical properties can be controllably tuned. In this work, we introduce interpenetrating polymer networks (IPN) hydrogels with improved elasticity due to a dual cross-linking mechanism in one of the networks. The proposed hydrogels contain entangled polymer networks of covalently cross-linked poly(ethylene glycol) methacrylate/diacrylate (PEGMA/PEGDA) and poly(vinyl alcohol) (PVA) with two types of physical cross-links—microcrystallites and tannic acid (TA). Rheological measurements demonstrate the synergistic enhancement of the elastic modulus of the single PEGMA/PEGDA network just upon the addition of PVA, since the entanglements between the two components are formed. Moreover, the mechanical properties of IPNs can be independently tuned by varying the PEGMA/PEGDA ratio and the concentration of PVA. Subsequent freezing–thawing and immersion in the TA solution of IPN hydrogels further increase the elasticity because of the formation of the microcrystallites and OH-bonds with TA in the PVA network, as evidenced by X-ray diffraction and ATR FTIR-spectroscopy, respectively. Structural analysis by cryogenic scanning electron microscopy and light microscopy reveals a microphase-separated morphology of the hydrogels. It promotes extensive contact between PVA macromolecules, but nevertheless enables the formation of a 3D network. Such structural arrangement results in the enhanced mechanical performance of the proposed hydrogels, highlighting their potential use for tissue engineering.

## 1. Introduction

Polymer hydrogels are among the most promising materials for tissue regeneration [1,2]. They consist of a cross-linked polymer network, swollen in water, and are ideal candidates for tissue repair because of their biomimetic structure, high water content, and cytocompatibility [3,4]. Hydrogels are introduced in the damaged tissues and serve as scaffolds for cell growth. During this process, gel matrices fulfill the structural function, replacing the damaged part. Huge efforts have been directed at the development of biomedicine-aimed hydrogels based on bio-sourced polymers [5], especially polysaccharides [6,7]. However, in some cases, their mechanical properties and temporal stability are insufficient: for instance, in the case of long-term regeneration of the connecting tissues, e.g., meniscus [8] and cartilage [9], which may last for several months. In this case, hydrogels formed by synthetic macromolecules are preferable [10], because they allow for a controlled variation in the mechanical properties of a wide range while preserving the stability of the material.

Different strategies have been established to enhance the mechanical properties of synthetic hydrogels [11]. For instance, combining several types of macromolecules into interpenetrating [12], double [13,14], or multiple [15,16] networks is a common approach to tune the elastic modulus, toughness, deformability, and other mechanical characteristics. There are several main factors influencing the behavior of such networks under the mechanical load: (1) entanglements between two networks, which are in many cases set by the concentration of each network and by the molecular weight of macromolecules [17]; (2) type and strength of cross-links in each network, and, in some cases, between the networks [18]; and (3) microstructure [19], which may be formed during or after the synthesis. Among these factors, changing the cross-linking strategy of each component of the interpenetrating networks (IPN) is a promising way to tune their mechanical properties, as it provides a possibility to explore the cross-links of different chemical natures and strengths. For such an approach, synthetic hydrogels are preferable over natural polymers, because they allow for easier incorporation of different types of cross-links, depending on the specific synthetic polymer.

One of the possible ways to tune the viscoelasticity of polymer networks is multiple cross-linking [20,21], which allows for independent variation in each cross-linker’s type and concentration. Different dual-cross-linked synthetic networks of a single polymer have been reported [20], including poly(acrylic acid) with covalent N,N′-methylenebisacrylamide cross-links and ionic bonds formed by Fe^3+^ ions [22], or PEG-based hydrogels with various cross-linkers [23], among others. In many of these systems, an increase in mechanical properties as compared to the single cross-linked networks was demonstrated. In recent years, dual cross-linking has been applied to single (one-component) poly(vinyl alcohol) (PVA) networks. For instance, PVA was covalently cross-linked by glutaric aldehyde and combined with physical cross-linking by borate ions [24,25] or microcrystallites [26], the latter being formed as a result of subsequent cycles of freezing and thawing [27]. A recently reported way of PVA cross-linking involves multiple hydrogen bonding with tannic acid (TA) [28,29]. Mechanically strong and tough PVA hydrogels with both TA and microcrystallites were described [30].

In this context, a suitable new way to tune the mechanical response of hydrogels consists of combining the interpenetrating network and a dual cross-linking strategy [31,32]. However, the factors governing the mechanical properties of such systems are not well understood. This is due to the increased complexity of the system, involving the formation of microstructures (for instance, microphase separation between two networks [33]), which largely influences the cross-linking of each network.

The aim of this work is to explore the influence of dual cross-linking and microstructure formation on the mechanical properties of IPNs, on a representative two-component system containing: (1) a network of poly(ethylene glycol) methacrylate (PEGMA) cross-linked by poly(ethylene glycol) diacrylate (PEGDA); and (2) a network of PVA dually cross-linked by microcrystallites and TA. For the first time, we employ cryogenic scanning electron microscopy (cryo-SEM) to such a system, in order to correlate the microphase formation with the rheological properties. Microphase separation increases the number of contacts between PVA macromolecules and allows for its tighter cross-linking both by microcrystallites and TA, while keeping the integrity of the 3D network. PVA cross-linking results in an increase in the elastic modulus of the hydrogels. At the same time, variation in the PEGMA/PEGDA cross-linking density allows for an independent tuning of the elastic modulus. The results of this work expand the ways to have a controlled variation in the mechanical properties of synthetic hydrogels. Both PEGMA/PEGDA [34] and PVA [35] networks are shown to be biocompatible, which provides a possibility of the interpenetrating network applications in biomedicine.

## 2. Materials and Methods

### 2.1. Materials

PEGDA (Mn = 575 g/mol, purity > 98%), PEGMA (Mn = 360 g/mol, purity > 98%), and TA (purity > 99%) were purchased from Sigma-Aldrich. PVA (average molecular weight 22,000 g/mol, purity > 98%) was obtained from Zhengzhou Alfa Chemical (China). Lithium 2,4,6-trimethylbenzoyl phosphinate (TPO-Li, purity > 97%) was bought from Merck (Darmstadt, Germany).

### 2.2. Sample Preparation

**Figure 1:**
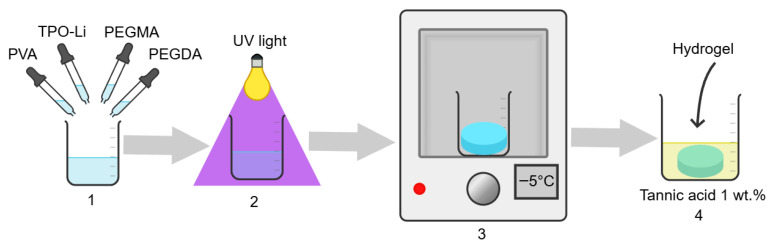
Scheme of the synthesis of hydrogels based on IPNs of PEGMA cross-linked by PEGDA, and PVA cross-linked with microcrystallites and TA: (1) Mixing PEGMA monomer, PEGDA cross-linker, TPO-Li initiator, and PVA in water solution. (2) Photo cross-linking of PEGMA/PEGDA network by UV light. (3) Cross-linking of PVA network with microcrystallites by freezing–thawing. (4) Additional cross-linking of PVA with TA.

The stages of obtaining the PEGMA/PEGDA/PVA hydrogels are presented in Figure 1. Dissolving 9 wt.% of PVA in water was performed according to the procedure described earlier [36]. The required quantities of PEGMA, PEGDA, and aqueous PVA solution were mixed, the volume was adjusted by Milli-Q water, and the mixtures were homogenized by a T10 basic ULTRA-TURRAX dispenser (IKA, Breisgau, Germany). Then, 0.25 wt.% of the photoinitiator TPO-Li was added into the system and mixed by a magnetic stirrer, and the solutions with a thickness of 5 mm were subjected to UV-irradiation at 365 nm (9 W intensity) for several minutes. The required time for photo cross-linking was estimated from the rheological measurements of viscoelasticity of the prepared PEGMA/PEGDA/PVA gels after 0.5; 1; 2; 5 and 10 min of exposure (Supplementary Figure S1). The dependence of the elastic modulus G_0_ of the prepared PEGMA/PEGDA/PVA gels on time of UV-irradiation is shown in Supplementary Figure S2. As more cross-links were formed in the gels, G_0_ increased with time, until a constant value was acquired. Therefore, in the following experiments, the photo cross-linking time was fixed at 10 min.

The concentrations of the monomer PEGMA and of the cross-linker PEGDA in the prepared gels varied from 5 to 11 wt.%. The concentration of PVA was changed within the range of 0–5 wt.%.

After preparation, the gels were frozen at a temperature of −5 °C for 2 h, and thawed until room temperature for 8 h, in order to form microcrystallites in the PVA network. For dual cross-linking, the gels (2 g) were submerged into 1 wt.% water solution of TA (50 mL) for 5 min to form additional bonds between PVA macromolecules, and then rinsed by distilled deionized water to remove the remains of the TA solution from the surface of the sample.

In the following text, the gels with x wt.% of PEGMA, y wt.% of PEGDA and z wt.% of PVA are denoted as follows: PEGMAx/PEGDAy/PVAz (for details see Supplementary Table S1). For the gels subjected to freezing and thawing, the following notation is used: PEGMAx/PEGDAy/f-PVAz. The gels submerged in the TA solution are referenced as PEGMAx/PEGDAy/f-PVAz/TA.

### 2.3. Rheology

The rheological measurements were performed using a shear stress rheometer Anton Paar MCR302 (Anton Paar, Austria) with a “plate-plate” measuring cell (bottom plate P-LP25/AL/G1; top plate PP25). The gap between the plates was equal to 5 mm. The dependences of the storage G′ and loss G″ moduli on the frequency of the applied stress ω were measured in the frequency range of 0.05–500 rad/s with the deformation amplitude of 0.1%, which corresponds to the linear viscoelasticity of the hydrogels (Supplementary Figure S3). The temperature was maintained at 20 ± 0.5 °C with Peltier elements.

### 2.4. Light Microscopy

The overall morphology of hydrogels at a lower resolution (few μm scale) was studied by light microscopy (LM), using the DM6000 M microscope (Leica, Austria) [37]. The thin sections for LM observations were prepared by cutting the samples with a sharp blade, while the samples were submerged in water. The thin sections were placed into a small water droplet between the standard support and cover glasses for light microscopy and observed in transmitted light mode (diascopic illumination).

### 2.5. Cryogenic Scanning Electron Microscopy

The morphology of hydrogels at a higher resolution (hundreds of nm scale) was studied by cryogenic low-vacuum scanning electron microscopy (cryo-LVSEM, or cryo-SEM). The experiments were carried out using a MAIA3 field emission scanning electron microscope (TESCAN), equipped with a secondary electrons (SE) detector and a Peltier cooling stage. The details of the experiments are described elsewhere [38]. Briefly, a small piece of the hydrogel sample (ca 2 × 2 × 2 mm^3^) was quickly frozen in liquid nitrogen and transferred to a Peltier Specimen Stage (cooled to −15 °C) in a vacuum chamber of the microscope. The frozen specimen was fixed on the Peltier specimen stage with a droplet of water (the water droplet freezes within one second, fixing the sample to the stage) and a thin upper layer of the specimen was cut off with a liquid nitrogen-cooled razor blade (this removes the upper layer of the frozen specimen, which can be deformed and/or covered with ice microcrystals). The morphology of the hydrogel was observed in a low-vacuum mode (chamber pressure 50 Pa), using a low-vacuum SE detector at an accelerating voltage of 15 kV. ImageJ software was used to construct the histogram of the microphase-separated domains sizes.

### 2.6. X-Ray Diffraction

The formation of the PVA microcrystallites was followed by X-ray diffraction (XRD) using a Bruker D8 Discover instrument with Cu Kα radiation (wavelength λ = 1.54 Å) at room temperature. In order to avoid the drying of the gels during acquisition, the gel was placed in a small Petri dish, located at the sample stage, and the bottom of the gel was immersed in water.

### 2.7. UV-VIS Spectroscopy

UV-VIS spectroscopy was employed to evaluate the amount of TA absorbed by the hydrogels after their immersion in TA solutions, as well as the leaching of the TA from the hydrogels.

To qualitatively evidence the presence of TA in the hydrogel, 0.05 g of PEGMA11/PEGDA5/f-PVA3/TA was dispersed in 10 mL of distilled water with an ultrasonic homogenizer Bandelin SONOPULS HD 4100 at 70 W for 30 min until a homogeneous suspension was prepared. The suspension was put into a 1 cm quartz cuvette. The UV-VIS spectrum of TA in the suspension was obtained using a UV-spectrometer Shimadzu UV-1800 in the range of 250–800 nm. A similarly prepared suspension of PEGMA11/PEGDA5/f-PVA3 gel without TA was used as a reference sample.

The release of TA from PEGMA11/PEGDA5/f-PVA/TA gel was studied in the following way. In the experiment, PEGMA11/PEGDA5/f-PVA3/TA hydrogel (2 g) was placed in a vial containing 50 mL of Phosphate-Buffered Saline (PBS) and kept at room temperature for 7 days. Every 24 h, 3 mL of the solution was withdrawn, and the UV-VIS spectra were recorded with a UV-spectrometer Shimadzu UV-1800. Pure PBS was used as a reference. To maintain a constant volume, the withdrawn PBS was returned to the vial after measurement.

### 2.8. Fourier-Tranform Infrared Spectroscopy

Attenuated total reflectance (ATR) FTIR-spectroscopy was applied to study the cross-linking of PVA with TA in the IPNs. The spectra were collected on an FSM 2202 spectrometer (OKB Spectr, Russian Federation) in the wavenumber range 4000–500 cm^−1^ with a resolution of 1 cm^−1^. To decrease the water residue, the hydrogels were freeze-dried for 24 h before the experiments.

### 2.9. Swelling

The swelling of the gels was evaluated by putting the gel pieces into a physiological saline (0.9 wt.% NaCl in water) and measuring the masses of gels over time *m*(*t*). The ratio of the initial gel’s mass (*m*_0_) to the outer medium mass was fixed to 1:100. The swelling coefficients were calculated as the following:

$$\alpha (t)=\;\frac{m\left(t\right)-m_{0}}{m_{0}}$$

### 2.10. Biocompatibility Analysis

MTT analysis was performed to evaluate the biocompatibility of hydrogels. It is a colorimetric method used to assess cell viability and proliferation, as well as to measure cytotoxicity. It is based on the ability of enzymes present in living cells to reduce tetrazolium salt (MTT) to an insoluble, purple-colored formazan. Human dermal fibroblasts were employed, which had been thoroughly characterized and previously published [39].

#### 2.10.1. Hydrogel Stock Buffers

For the basic assessment of the hydrogels’ biocompatibility, the stock PBS buffer was put in contact with the hydrogels, and then the effect of this buffer on the cell viability was evaluated. The experiment models the contact of biofluids with the hydrogel inside the body. Hydrogels with a mass of 2 g were put in 2 mL of PBS buffer for 1 week. Then, 100 µL of this PBS buffer was taken for the MTT analysis.

For this analysis, fibroblasts were cultured in standard DMEM (Dulbecco’s Modified Eagle Medium), supplemented with 10% fetal bovine serum (FBS) and 1% antibiotic-antimycotic solution (penicillin-streptomycin). The cells were kept at 37 °C in a 5% CO_2_ atmosphere in a humidified environment. Once the cells reached a certain surface density, they were removed from the flasks with a trypsin–EDTA solution, resuspended in fresh medium, and counted using an automatic cell counter. Fibroblasts were seeded in 96-well plates at 7000 cells per well. After 12–24 h of incubation, which was necessary for cell attachment, the medium was replaced with fresh medium. In the experimental series, the initial PBS buffer probe, as well as sequential dilutions of 1:2, 1:4, 1:8, and 1:16, were added to the prepared well plates. The plates were incubated under standard conditions for cell interaction with the test substances. Cytotoxicity was assessed using the CyQUANT™ MTT Cell Proliferation Assay Kit (Invitrogen). On the day of the experiment, a 12 mM MTT solution (5 mg/mL PBS) and an SDS-HCl solution (1 g SDS per 10 mL 0.01 M HCl) were prepared. After treatment with the samples, the culture medium in the plates was replaced with fresh medium (100 μL per well), then 10 μL of MTT solution was added. Negative control wells contained medium without gels, with the addition of MTT. The plates were incubated for 4 h at 37 °C in a 5% CO_2_ atmosphere. After incubation, 100 μL of SDS-HCl solution was added to each well to dissolve the formazan, and the plates were left at 37 °C for 4–18 h. Optical densities (OD) were measured using a Multiskan Sky High spectrophotometer (Thermo Fisher Scientific, USA) at a wavelength of 570 nm. The obtained values were used to calculate cell viability in comparison with the negative control according to the equation:

$$Cell\;viability=\;\frac{{OD}_{sample}}{{OD}_{negative\;control}}\cdot 100\%$$

#### 2.10.2. Hydrogels in Direct Contact with Cells

To further evaluate the biocompatibility of hydrogels, they were put in direct contact with the fibroblasts within the MTT test. Cells were cultivated in DMEM with glutamine and 4 g/l glucose (PanEco, Russia), with the addition of 10% fetal calf serum (Biolot, Russia) and 1% penicillin and streptomycin antibiotics (PanEco, Russia). Cells were cultured in 24-well plates (TPP, Switzerland) (1 mL volume) at a concentration of 1.4 × 10^5^ cells/mL at 37 °C in a 5% CO_2_ atmosphere. A nutrient medium was used as a negative control, with 4 replicates. As a positive control, 10% dimethyl sulfoxide (DMSO, PanEco, Russia) was added to the cells. The plates were incubated until 80% confluence of the cell monolayer was achieved. Then, 2 × 2 × 2 mm^3^ gel samples were added to the plate wells, with 4 replicates. The samples were pre-sterilized by UV treatment for 30 min. After adding the samples, the cells were incubated for 2 days with visual monitoring of cytopathic effects. Then, a 0.5% solution of MTT dye (Sigma-Aldrich, USA) was added to each well in a volume of 1:10. The plates were incubated under the same conditions (5% CO_2_, 37 °C) for 4 h. Then, the supernatant was carefully removed from the wells and 400 μL of dimethyl sulfoxide was added to dissolve the formazan precipitate that had formed at the bottom of the wells. For more complete dissolution of the formazan, the plates were shaken for 20 min at 250 rpm at room temperature on an orbital thermostatic shaker (ELMI, Latvia). The optical density (OD) was recorded at a wavelength of 595 nm on a multimode microplate spectrophotometer (Biobase, China).

## 3. Results and Discussion

### 3.1. Rheological Properties and Microstructure

**Figure 2:**
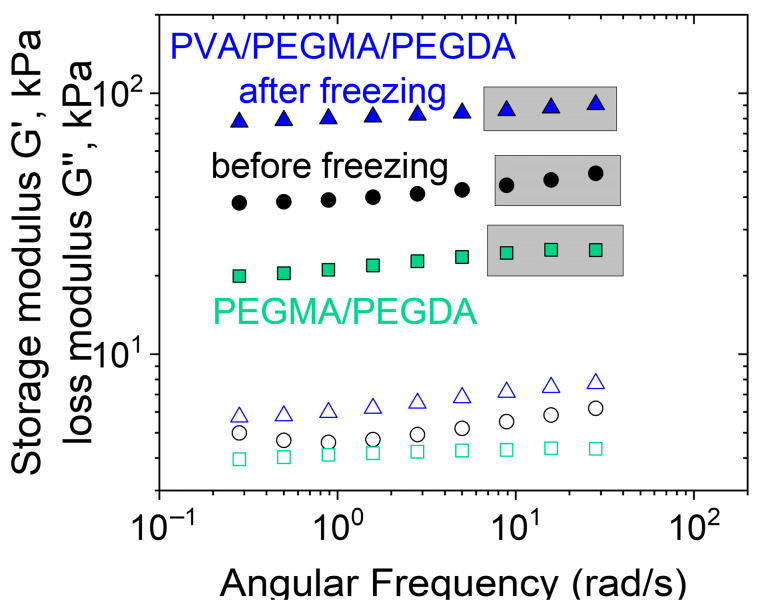
Frequency dependences of the storage G′ (filled symbols) and G′′ (open symbols) for PEGMA11/PEGDA11 (squares); PEGMA11/PEGDA11/PVA3 hydrogel (circles); and PEGMA11/PEGDA11/f-PVA3 (triangles) hydrogels. Temperature: 20 °C.

Figure 2 shows the frequency dependencies of the storage G′ and loss G″ moduli for the PEGMA11/PEGDA11 and PEGMA11/PEGDA11/PVA3 hydrogels. In both cases, G′(ω) > G″(ω) for all studied frequencies ω, and a well-defined plateau is observed in the G′(ω) curves. It confirms the formation of the photopolymerized PEGMA/PEGDA network, both in the absence and in the presence of PVA. The shear elastic modulus G_0_ (plateau value of G′) of the two-component network is higher than that of the single PEGMA/PEGDA network, which is due to the entanglements between PVA and PEGMA macromolecules, resulting in the formation of a semi-interpenetrating structure in the PEGMA11/PEGDA11/PVA3 hydrogel [40]. After a cycle of freezing—thawing, the gel-like behavior of the PEGMA11/PEGDA11/PVA3 is predictably conserved (Figure 2): G′(ω) > G″(ω), and the plateau of G′(ω) is observed at higher ω. Moreover, the elastic modulus G_0_ of the frozen—thawed gel is circa 2 times higher than that of the gel before freezing. It points out the changes in the gel’s microstructure while freezing–thawing, which is analyzed below.

**Figure 3:**
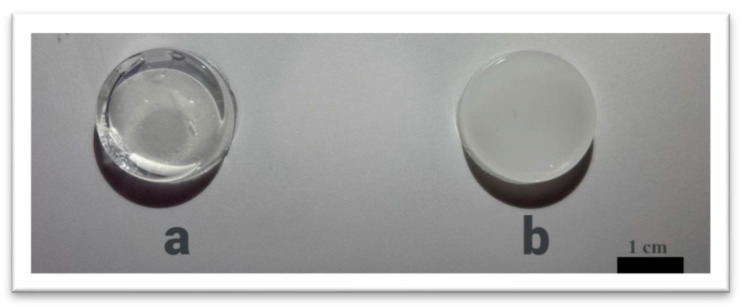
Macrophotographs of the PEGMA11/PEGDA11 (**a**), and PEGMA11/PEGDA11/f-PVA3 (**b**) hydrogels.

The PEGMA/PEGDA/f-PVA hydrogels were visually compared to hydrogels with the corresponding single photo cross-linked PEGMA/PEGDA network. The single network hydrogel is macroscopically homogeneous and transparent (Figure 3a). By contrast, the frozen–thawed two-component hydrogel, containing 3 wt.% of PVA, is opaque and almost non-transparent (Figure 3b); this indicates the formation of micro-inhomogeneities.

**Figure 4:**
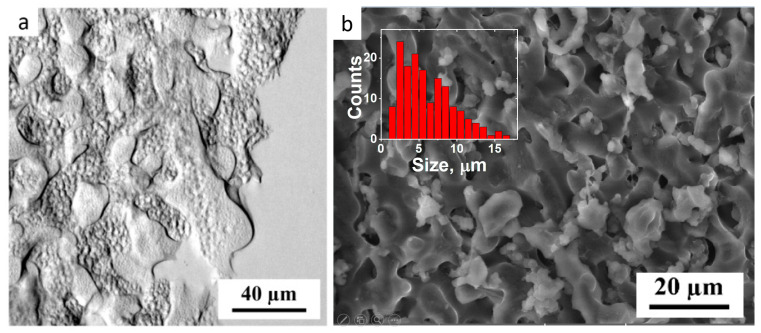
Light microscopy (**a**), and cryo-SEM (**b**) micrographs of the PEGMA11/PEGDA11/*f*-PVA3 hydrogel inset in Figure 4b shows the size distribution of the microstructures seen in the micrograph.

The observed inhomogeneities arise from two sources. The first type is related to the microphase separation between the components of IPN. This is evidenced by LM of the PEGMA/PEGDA/PVA gel (Figure 4a). In the micrograph, “smooth” and “grainy” micron-sized inhomogeneities (microphases) can be observed. Cryo-SEM confirms the two-phase hydrogel microstructure, demonstrated by LM (Figure 4b). It shows a microphase-separated rough co-continuous structure, formed by lighter and darker interpenetrating inhomogeneities, with a mean size of circa 5 µm, as determined from the histogram of the microstructures’ sizes (inset in Figure 4b). Similar microphase-separated structures were observed for several water-soluble polymer mixtures [41], but it has not been evidenced in the literature for the systems containing a freeze–thawed PVA network.

**Figure 5:**
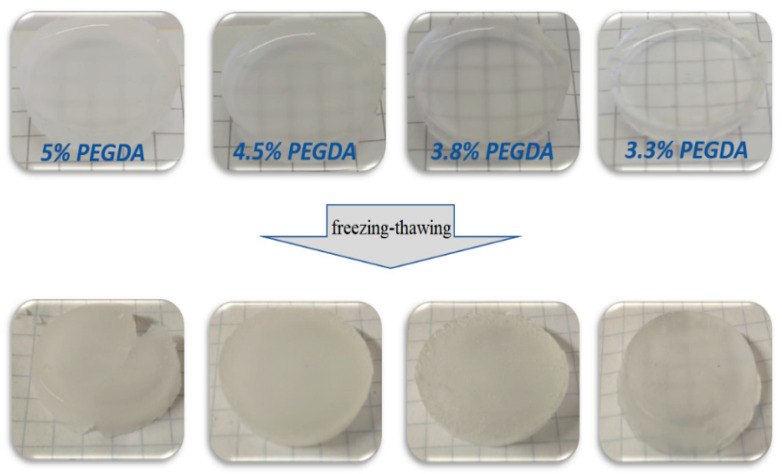
Macrophotographs of PEGMA/PEGDA/PVA hydrogels containing various concentrations of PEGDA before (up) and after (bottom) freezing–thawing. Concentrations of PEGMA and PVA are equal to 11 and 3 wt.%, respectively. The horizontal dimension of the background squares is 5 mm.

Note that the hydrogels with a tighter cross-linked PEGMA/PEGDA network (e.g., containing more of the cross-linker, PEGDA) become opaque before freezing–thawing (Figure 5). This means that the microphase separation occurs before the cross-linking of PVA. It is worth mentioning that the opacity of the gels increases with the concentration of PEGDA, evidencing a stronger tendency for microphase separation of the tightly cross-linked PEGMA/PEGDA network from the PVA network. The reason for such microphase separation is presumably the following. As water is known to be a θ-solvent for PVA at room temperature [42], the contact of PVA macromolecules with each other is as favorable as with the solvent. The formation of a PEGMA/PEGDA network reduces the conformational entropy of the PVA chains, due to their mutual entanglements. These topological restrictions become more pronounced when the PEGMA/PEGDA is cross-linked tighter. Note that before freezing–thawing, the macromolecules of PVA are rather mobile (due to a low molar weight). Therefore, the observed loss in conformational entropy upon cross-linking of PEGMA with PEGDA is sufficient. As a result, it is favorable for PVA to form a separate microphase. Afterwards, freezing–thawing “fixes” PVA molecules and additionally reduces their mobility in the microphase-separated structure.

**Figure 6:**
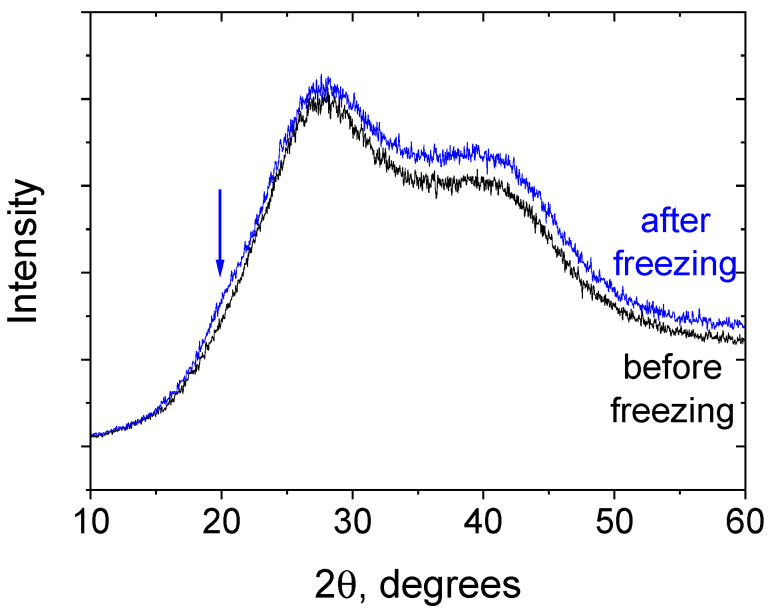
X-ray diffraction patterns of the PEGMA11/PEGDA11/PVA3 (black) and PEGMA11/PEGDA11/f-PVA3 (blue). Temperature: 20 °C. Arrow points out an additional XRD signal after freezing and thawing of the gel.

The second type of micro-inhomogeneities in the PEGMA/PEGDA/PVA networks are microcrystallites formed by the PVA macromolecules after freezing–thawing. Their formation is evidenced by X-ray diffraction (Figure 6), which shows the intensity increase in the range of 18–23 degrees, occurring after freezing–thawing and corresponding to PVA crystalline packing [43]. The formation of the additional cross-links between macromolecules of PVA accounts for the observed increase in G_0_ (Figure 2). This explanation seems to be reasonable, since, in the literature, PVA forms microcrystallites upon freezing–thawing, in the absence of PEGMA/PEGDA network, resulting in similar opaque hydrogels [44]. In our system, the microphase separation with the PEGMA/PEGDA network favors the crystallization of PVA, because it enhances the amount of contact that the PVA macromolecules have with each other. Note that, in our work, PVA with a low molecular weight (22,000 g/mol) is employed, and it does not form hydrogels via freezing–thawing in the concentration range studied in the article (0–5 wt.%). It happens because these concentrations are below or very close to the overlap concentration (C* = 3 wt.%) and are much lower than the entanglement concentration, which is usually 5–10 times higher than C*. Therefore, there are not enough mutual contacts between PVA macromolecules, so no cross-links can be formed by microcrystallites.

However, when the PVA concentration is sufficiently higher, freezing–thawing results in gelation (an example of a 20 wt.% PVA gel formed by freezing–thawing is shown in Supplementary Figure S4). Thus, PEGMA/PEGDA favors PVA crystallization in a way that PVA is concentrated in its microphase-separated domains. Increase in the local PVA concentration in these domains results in an increase in the number of PVA–PVA contacts, favoring the formation of microcrystallites.

Therefore, PEGMA/PEGDA/f-PVA IPN hydrogels exhibit a microphase-separated morphology. Increase in the mechanical properties of the IPNs, as compared to the single PEGMA/PEGDA network, is related to additional topological entanglements between PVA macromolecules and PEGMA chains, as well as to the additional cross-linking of PVA chains by microcrystallites, formed upon freezing–thawing.

### 3.2. Effect of PEGMA and PEGDA Concentrations

**Figure 7:**
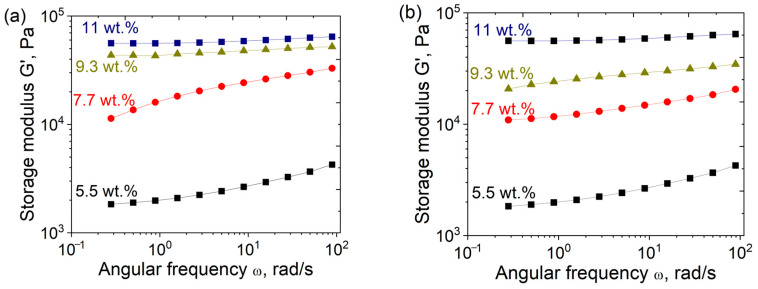
Frequency dependencies of the storage modulus (G′) for PEGMA/PEGDA11/f-PVA3 hydrogels with different contents of PEGMA (**a**), and PEGMA11/PEGDA/f-PVA3 hydrogels with different contents of PEGDA (**b**). Temperature: 20 °C.

In order to determine the factors with the most pronounced influence on the elasticity of PEGMA/PEGDA/f-PVA hydrogels, the dependencies G′(ω) were evaluated for gels with different PEGMA and PEGDA contents at a fixed concentration of PVA (3 wt.%). Higher concentrations of PEGMA and PEGDA lead to more plateau-like G′(ω) curves (Figure 7). This demonstrates the formation of a tighter polymer network [45].

**Figure 8:**
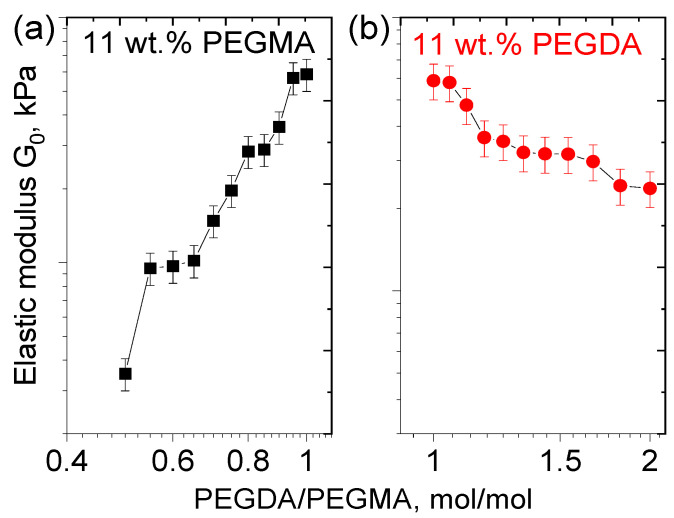
Dependencies of the elastic modulus G_0_ on the PEGDA/PEGMA molar ratio in the PEGMA11/PEGDA/f-PVA3 (**a**); and PEGMA/PEGDA11/f-PVA3 hydrogels (**b**). Temperature: 20 °C. The lines connecting experimental points serve to guide the eye.

At a fixed amount of PEGMA, G_0_ of PEGMA/PEGDA/f-PVA hydrogels rises with the increasing PEGDA concentration (Figure 8a), because PEGDA serves as a cross-linker in the PEGMA/PEGDA network and induces a tighter cross-linking of the network. Since this effect is also observed in the presence of the PVA network, cross-linked by microcrystallites, the first PEGMA/PEGDA network preserves and contributes to the elasticity of the IPN. At the same time, at a fixed amount of PEGDA, a slight decrease in G_0_ with an increasing concentration of PEGMA is observed (Figure 8b), since PEGMA serves as a monomer in the PEGMA/PEGDA network. This behavior is due to the fact that the increasing PEGMA concentration leads to the increased length of sub-chains in the PEGMA/PEGDA network, but does not enhance the number of cross-links. This effect was previously reported for PEGMA/PEGDA hydrogels in the absence of PVA [46].

Therefore, the concentration of the first network cross-linker PEGDA has the most pronounced influence on the elasticity of the IPN hydrogels and allows for tuning the elastic modulus at a fixed concentration of PVA. The effect of the PVA concentration on the elasticity is investigated below.

### 3.3. Effect of PVA Concentration and TA Addition

**Figure 9:**
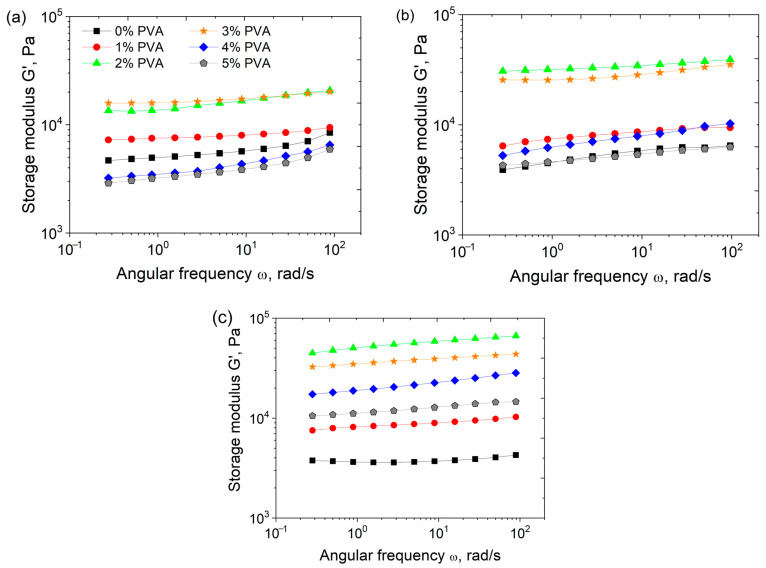
Frequency dependences of the storage modulus G′(ω) of PEGMA11/PEGDA5/PVA (**a**), PEGMA11/PEGDA5/f-PVA (**b**), and PEGMA11/PEGDA5/f-PVA/TA hydrogels (**c**), containing different amounts of PVA at 20 °C.

Frequency dependencies of the storage modulus G′(ω) for hydrogels PEGMA11/PEGDA5/PVA with various amounts of PVA are presented in Figure 9a. Increasing the PVA concentration from 0 to 5 wt.% leads to a non-monotonic behavior of the elastic modulus, G_0_ (Figure 9). G_0_ reaches a maximum at 2 wt.% PVA. This effect may be explained as follows: with the growth of PVA concentration, an increase in the number of entanglements in the IPN occurs; this promotes the formation of a denser network and an increase in the elastic modulus. At PVA concentrations higher than 2 wt.%, the formation of a microphase-separated structure takes place, leading to larger numbers of PVA–PVA and PEGMA–PEGMA contacts and lower values of G_0_. Note that PEGDA11/f-PVA3 hydrogel without any PEGMA contains only one “grainy” phase, and no microphase separation occurs (Supplementary Figure S5). This means that PEGMA may be primarily responsible for the microphase separation in the PEGMA/PEGDA/PVA hydrogels. This is due to a particular structure of the PEGMA/PEGDA network, in which the short-chain PEG segments of PEGMA serve as “side” groups of the polymer. The PEGMA/PEGDA copolymer can be regarded as a “brush” with very short PEG side chains, and there is a rather strong interaction between these PEG side chains of various macromolecules [47], leading to their concentration in a separate microphase. The non-monotonic behavior of G_0_ with the increasing PVA concentration also remains for PEGMA/PEGDA/f-PVA hydrogels (Figure 9b), but the corresponding absolute values of G_0_ of frozen-thawed hydrogels are higher at almost all the PVA concentrations. This proves the contribution of microcrystallite cross-links to the elasticity of IPN.

Submerging the PEGMA/PEGDA/f-PVA hydrogels into 1 wt.% aqueous solution of TA leads to a further increase in G_0_ (Figure 9c) at various PVA concentrations, attributed to the formation of additional cross-links due to hydrogen bonds between hydroxyl groups of PVA and phenol groups of TA [28]. In the UV-VIS spectrum of the PEGMA11/PEGDA5/f-PVA/TA gel (cut into small pieces and dispersed in water, Supplementary Figure S6), one can observe a peak at 271 nm, characteristic of TA [48], which proves the fact that TA enters into the gel. Using UV-VIS spectra of TA water solutions of different concentrations (Supplementary Figure S7), TA concentration in the gel is semi-quantitatively estimated to be equal to 0.9 wt.%.

**Figure 10:**
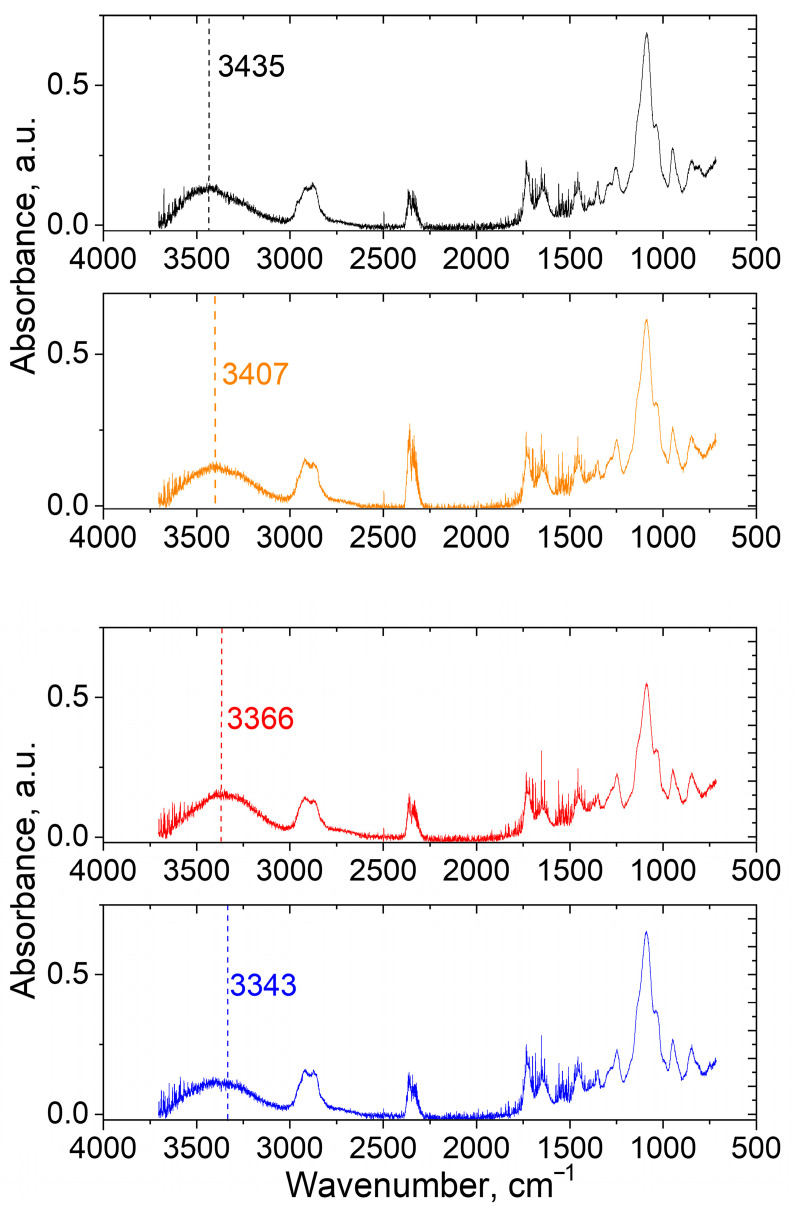
ATP FTIR spectra of PEGMA11/PEGDA5 hydrogel (black); PEGMA11/PEGDA5/PVA3 hydrogel (orange); PEGMA11/PEGDA5/f-PVA3 hydrogel (red) and PEGMA11/PEGDA5/f-PVA3/TA hydrogel (blue).

To provide direct evidence of H-bonds formation, ATP FTIR spectra of the PEGMA11/PEGDA5; PEGMA11/PEGDA5/PVA3; PEGMA11/PEGDA5/f-PVA3 and PEGMA11/PEGDA5/f-PVA3/TA hydrogels were received (Figure 10). In the spectra, typical signal of OH-groups at 3000–3700 cm^−1^ [29,30], can be clearly observed (Figure 10). After the addition of 3 wt.% of PVA to the PEGMA11/PEGDA5 hydrogel, the maximum of the peak moves from 3435 to 3407 cm^−1^. Cross-linking of the PEGMA11/PEGDA5/PVA3 with microcrystallites and TA results in a consistent shift in the maximum to 3366 and 3343 cm^−1^, respectively. In the literature, a similar shift points out the reduction in force constants of the chemical bonds, caused by intra- or intermolecular H-bonding [29,30]. This result proves the formation of a dually cross-linked network of PVA (both by microcrystallites and by TA) within the IPNs of PEGMA11/PEGDA5/PVA3.

For biomedical application of the proposed hydrogels, no leaching is important. The possible leaching was measured with UV-VIS spectroscopy. The UV-VIS spectra of the probes of the PBS with the immersed PEGMA11/PEGDA5/f-PVA/TA gel, sequentially collected for 7 days, are depicted in Supplementary Figure S8. The values of the absorbance of the probes measured at the wavelength of 271 nm coincide with each other and practically do not differ from the baseline. Therefore, the released amount of TA is insignificant within the experimental error, probably due to strong H-bonding [28,29,30].

**Figure 11:**
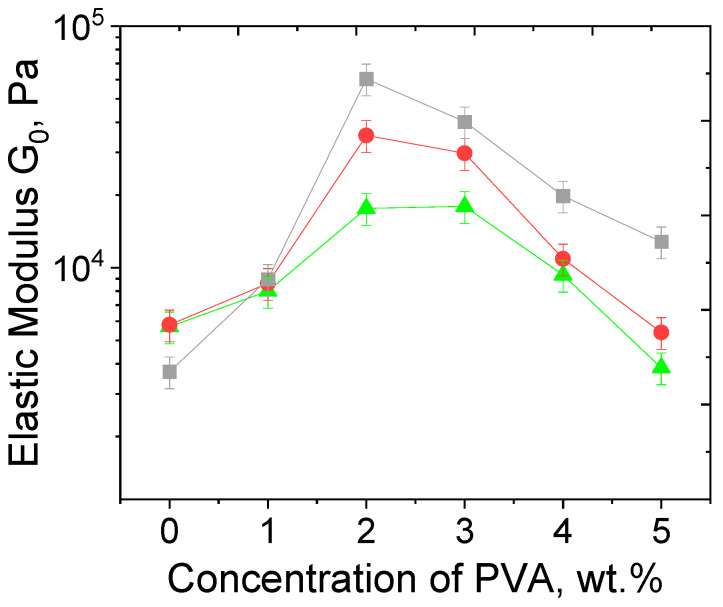
Dependencies of the elastic modulus G_0_ on PVA concentration in PEGMA11/PEGDA5/PVA (triangles), PEGMA11/PEGDA5/f-PVA (circles), and PEGMA11/PEGDA5/f-PVA/TA (squares) hydrogels. Temperature: 20 °C. The lines connecting experimental points serve to guide the eye.

The relative growth of G_0_, induced by dual cross-linking, becomes more prominent upon the addition of more than 2 wt.% of PVA, since a tighter PVA network is formed in the hydrogels when the networks are microphase-separated. Note that the amount of PVA, which corresponds to the maximum of G_0_ in dual cross-linking hydrogels, remains the same as for those before freezing–thawing and the addition of TA (Figure 11). This proves that microphase separation of PVA in the PEGMA/PEGDA/f-PVA/TA hydrogels is independent from the formation of microcrystallites and hydrogen bonds with TA, and is just governed by the concentrations of PEGDA and PEGMA. This effect is to be expected, because the microphase-separated structure is already formed during the formation of the PEGMA/PEGDA network, in the presence of PVA. Further cross-linking of PVA by microcrystallites and TA proceeds in the microphase-separated structure.

**Figure 12:**
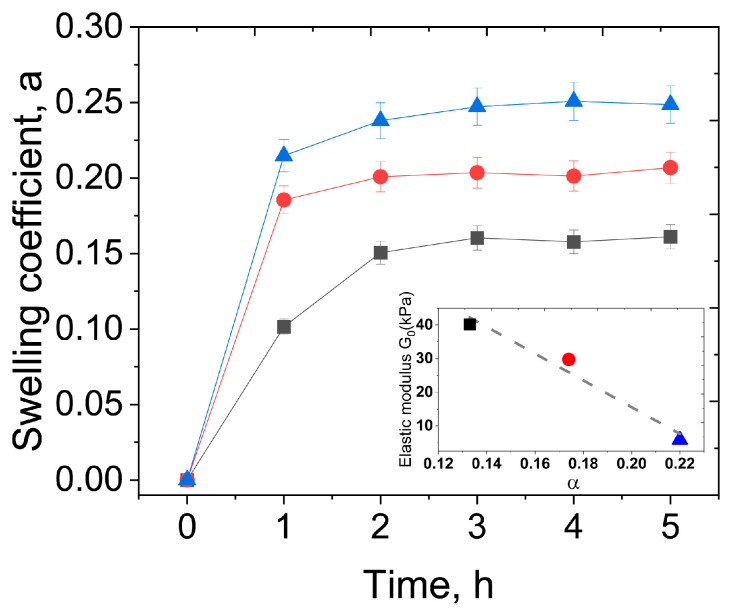
Time dependencies of swelling coefficients α of PEGMA11/PEGDA5 (blue); PEGMA11/PEGDA5/f-PVA3 (red); and PEGMA11/PEGDA5/f-PVA3/TA (black) hydrogels. The inset is the dependence of the corresponding values of elastic modulus G_0_ vs. α. The lines connecting experimental points serve to guide the eye.

For the practical application of the developed hydrogels as biomaterials, their swelling properties in the physiological saline were evaluated (masses of gels vs. time during swelling are shown in Supplementary Figure S9, and the swelling coefficients (relative mass gain) are depicted in Figure 12). The equilibrium swelling coefficients are rather small and are in the range of 0.1–0.25, meaning that the gels gain 10–25% of their initial weight. This is consistent with the uncharged nature of the hydrogels that are known to have low swelling coefficients, as compared to the polyelectrolyte gels [49]. The α values for the single PEGMA/PEGDA network align with the literature data [50]. Similarly, low values of the swelling coefficients were reported for the freeze–thawed single network PVA hydrogels [51]. Low-swelling hydrogels are preferable for tissue engineering applications, since they maintain structural integrity and mechanical properties by minimizing water absorption, and also prevent the compression of surrounding tissues when inserted in the body.

A clear correlation between the swelling coefficient and the elastic modulus of the hydrogels is seen; swelling is more pronounced for the gels with lower elasticity (inset in Figure 12). This is consistent with the fact that, according to the classical theory of network elasticity, G_0_ is proportional to the number density of cross-links *ν* [52,53]:

$$G_{0}∼0.5\;f\nu RT,$$

where f is the functionality of the cross-linker, e.g., the number of sub-chains which the cross-linker connects (4 in case of PEGDA), R is the universal gas constant, and T is temperature. At the same time, swelling coefficients are lower for tighter cross-linked gels [54]. Therefore, the swelling experiments support the formation of cross-links in the PVA network, both by microcrystallites and TA, since the introduction of these cross-links contributes to the increase in the elastic modulus, accompanied by the suppression of the gels’ swelling.

### 3.4. Biocompatibility

**Figure 13:**
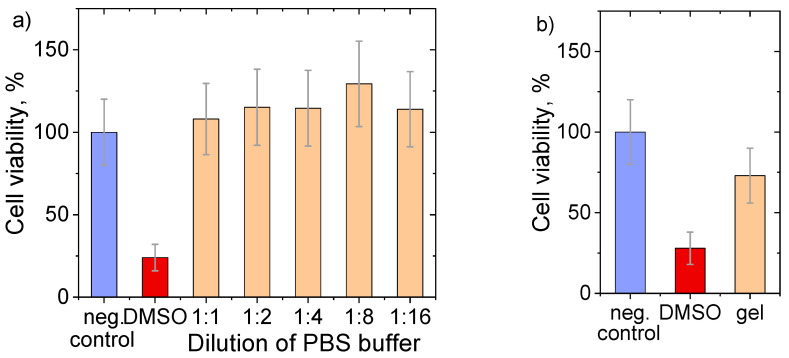
Results of MTT analysis showing cell (fibroblast) viabilities for: (**a**) dilutions of PBS buffer, which was previously put in contact with the hydrogel; (**b**) hydrogel directly put in contact with the cells. Cell viabilities were referenced to negative control; and 10 wt.% DMSO was used as a positive control. Gel’s composition: 11 wt.% PEGMA, 5 wt.% PEGDA, 3 wt.% PVA, freeze/thawed and cross-linked with TA.

Rheological and structural studies revealed that the optimal compositions of IPN hydrogels dually cross-linked by freezing–thawing and TA. To demonstrate the possibility of biomedical applications, biocompatibility testing on a model of dermal fibroblasts was carried out. Figure 13a shows the cell viability results, when the cells were put in contact with stock buffer solutions, in which the gels were previously immersed for one week. It is seen that the cells’ proliferation for the initial buffer and all its dilutions is stable and close to one of the controls, within the experimental error. This model experiment shows that the hydrogel is not cytotoxic (e.g., does not release any toxic substances), when put in contact with a biofluid.

When the hydrogel is put directly in contact with the cells, cell viability is above 70% (Figure 13b). According to the ISO 10993-5 standard test for in vitro cytotoxicity [55], the hydrogel is considered non-toxic. Figure S10 shows the cell morphology in the negative control experiment (a); positive control, e.g., contact of cells with 10% DMSO (b); and in direct contact with the gel (c). It is seen that the fibroblasts keep their elongated shape and do not change the morphology in the presence of the gel. By contrast, the addition of DMSO shows an obvious toxic effect—the cell morphology is “collapsed”, and no living fibroblasts are visible. These results show that the developed hydrogels possess sufficient biocompatibility for potential biomedical applications.

## 4. Conclusions

A new type of hydrogel with two IPNs of PEGMA, photo cross-linked with PEGDA and PVA, and dually cross-linked with microcrystallites and TA, has been synthesized. The incorporation of PVA is shown to increase the elastic modulus G_0_ of the hydrogels, due to the formation of additional topological entanglements between PVA chains and the PEGMA/PEGDA network. This effect is enhanced by consequent cross-linking of PVA chains with microcrystallites during freezing–thawing and cooperative hydrogen bonds after the addition of TA. The formation of microcrystallites and H-bonds between PVA macromolecules in IPN is evidenced by X-ray diffraction and ATR FTIR spectroscopy, respectively. The cross-linking of PVA is favored by the PEGMA/PEGDA network due to the microphase separation between the components and the formation of PVA-rich microdomains with an increased number of PVA–PVA contacts. This aligns with an increase in the rheological properties of various mixtures, due to microphase separation observed in the literature [56,57]. We show that dual cross-linking of PVA in IPN hydrogels with microcrystallites and TA is followed by an increase in the elastic modulus by factors of two and six, respectively. The elastic modulus of the dually cross-linked PEGMA/PEGDA/PVA hydrogel is close to that of a human’s muscles and tendons and somewhat smaller than cartilage [58]. Variation in the molar weight of PVA can be a possible way to improve its elasticity and will be a topic of the future paper. The prepared hydrogels were shown to be biocompatible by testing on a model of dermal fibroblasts. Thus, the developed hydrogels represent a suitable material for tissue regeneration strategies.

## Data Availability

The original contributions presented in this study are included in the article. Further inquiries can be directed to the corresponding author.

## Supplementary Materials

The following supporting information can be downloaded at https://www.mdpi.com/article/10.3390/polym17202737/s1: (1) Effect of UV-irradiation; (2) Hydrogel notation; (3) Determination of the linear viscoelasticity range; (4) Figure S4 PVA gel in the absence of PEGMA/PEGDA; (5) Figure S5 LM micrograph of PEGDA11/f-PVA3. hydrogel; (6) Amount of TA in the gels; (7) Leaching of TA from the gels; (8) Additional swelling data; (9) Additional biocompatibility data; (10) Estimation of the experimental errors.

## Abbreviations

The following abbreviations are used in this manuscript:

PEGMA	poly(ethylene glycol) methacrylate
PEGDA	poly(ethylene glycol) diacrylate
PVA	poly(vinyl alcohol)
TA	tannic acid
TPO-Li	lithium 2,4,6-trimethylbenzoyl phosphinate
LM	light microscopy
cryo-VSEM	cryogenic low-vacuum scanning electron microscopy
cryo-SEM	cryogenic scanning electron microscopy
FTIR	Fourier-transform infrared spectroscopy
