# Supplementary material for: Biocompatible Interpenetrating Network Hydrogels with Dually Cross-Linked Polyol
Source: Polymers (Basel). 2025 Oct 13;17(20):2737. doi: 10.3390/polym17202737 (PMC12567005; doi:10.3390/polym17202737)
Supplement: Supplementary file 1 [file polymers-17-02737-s001.zip › polymers-3866137-supplementary.pdf]

Biocompatible Interpenetrating Network Hydrogels with Dually Cross-Linked Polyol

Ulygbek B. Tuleuov[1], Alexander L. Kwiatkowski[2,*], Akerke T. Kazhmuratova[1], Lyazzat Zh. Zhaparova [1], Yermauyt Nassikhatuly [1], Miroslav Šlouf [3], Andrey V. Shibaev[4,*], Viktor I. Petrenko[4,5], Senentxu Lanceros-Méndez[4,5], Yerkeblan M. Tazhbayev[1]

AFFILIATIONS

[1]Faculty of Chemistry, Karaganda Buketov University, 100028 Karaganda, Kazakhstan

[2]Physics Department, Lomonosov Moscow State University, 119991 Moscow, Russia

[3]Institute of macromolecular chemistry, 16200 Prague, Czech Republic

[4]BCMaterials, Basque center for materials, applications and nanostructures, UPV/EHU Science Park, 48940 Leioa, Spain

[5]IKERBASQUE, Basque Foundation for Science, 48009 Bilbao, Spain

[*)]**Authors to whom correspondence should be addressed: kvyatkovskij@physics.msu.ru, andrey.shibaev@bcmaterials.net**

SUPPLEMENTARY INFORMATION

1. EFFECT OF UV-IRRADIATION. To evaluate the mechanical properties of PVA/PEGMA/PEGDA hydrogels after different times of photopolymerization, rheological measurements were performed. Figure S1 represents the corresponding frequency sweep curves of the storage modulus G′ and the loss modulus G″ in the range from 0.1 to 100 rad/s. In all cases, the storage modulus *G′* is larger than the loss modulus *G″*, independently of the applied frequency. It indicates a predominantly elastic behavior of the material, even after a short period of UV-irradiation. The dependencies *G′(ω)* exhibit a plateau at low frequencies, suggesting the formation of a hydrogel with the cross-linked polymer network. The plateau value of *G′* is the elastic modulus of the hydrogels G_0. Figure S2 shows the variation in the elastic modulus as a function of UV irradiation time.

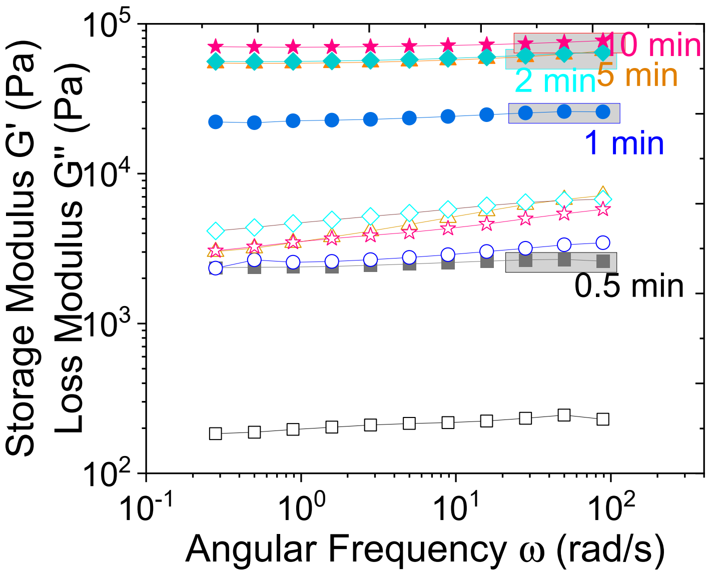

Figure S1. Frequency sweep curves of the storage G' (filled symbols) and loss G'' (empty symbols) moduli of the photo cross-linked PVA/PEGMA/PEGDA hydrogels after different periods of UV-irradiation (present in the figure). The plateau of G'(ω) curves corresponding to G_0 are marked with gray rectangles.

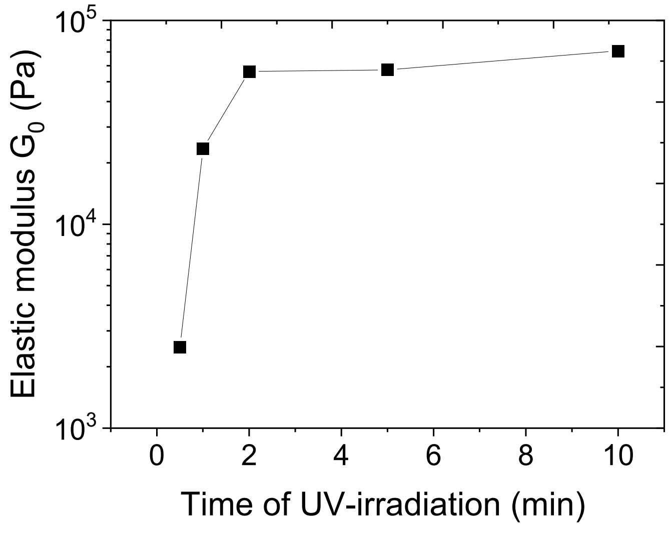

Figure S2. Elastic modulus G_0 of the photo cross-linked PEGMA/PEGDA/PVA hydrogels vs. period of UV-irradiation.

2. HYDROGEL NOTATION

Table S1. Notations of the prepared hydrogels of PEGMA, PEGDA and PVA cross-linked with microcrystallites and TA.

Notification	PEGMA (x wt.%)	PEGDA (y wt.%)	PVA (z. wt%)	Freezing –thawing	Tannic acid
PEGMAx/ PEGDAy	x	y	0	-	-
PEGMAx/ PEGDAy/ PVAz	x	y	z	-	-
PEGMAx / PEGDAy / f-PVAz	x	y	z	+	-
PEGMAx / PEGDAy / f-PVAz / TA	x	y	z	+	+

3. DETERMINATION OF THE LINEAR VISCOELASTICITY RANGE

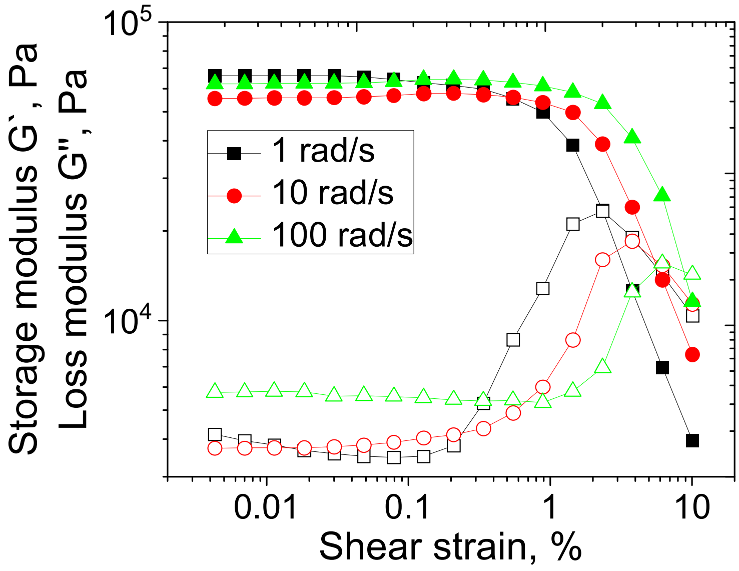

Figure S3. Dependencies of storage G′ (filled symbols) and loss G″ (empty symbols) on strain measured at 1; 10 and 100 rad/s for PEGMA11/PEGDA5/f-PVA3/TA hydrogel.

4. PVA GEL IN THE ABSENCE OF PEGMA/PEGDA

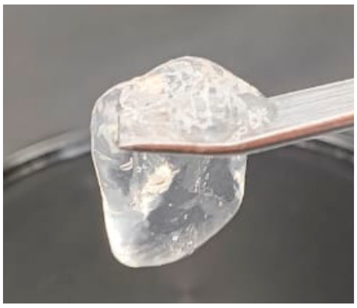

Figure S4. Freeze–thawed 20 wt% PVA hydrogel, prepared in the absence of PEGMA and PEGDA

5. LM OF PEGDA11/PVA2 HYDROGEL IN THE ABSENCE OF PVA

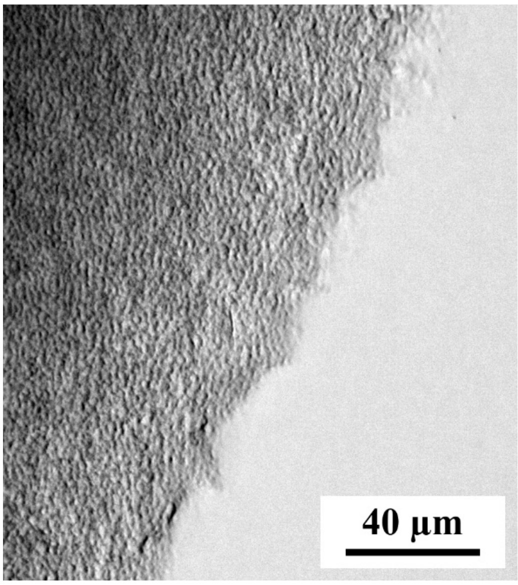

Figure S5. LM micrograph of the PEGDA11/f-PVA3 hydrogel.

6. AMOUNT OF TA IN THE GELS

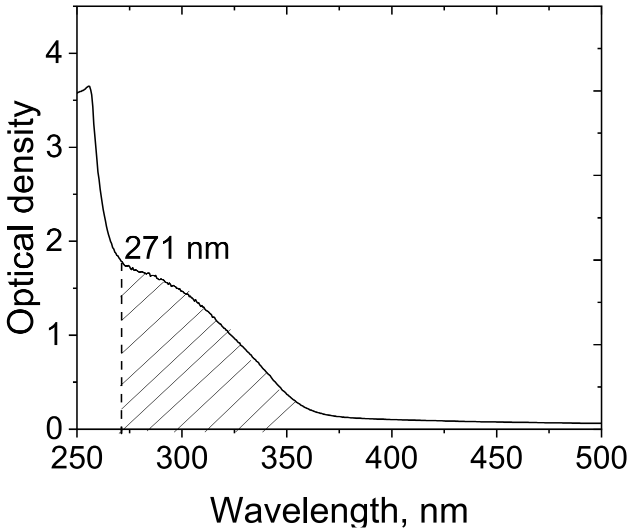

Figure S6. UV-VIS spectrum of PEGMA11/PEGDA5/f-PVA3/TA hydrogel. The dashed lines indicate the right shoulder of TA peak with maximum of 271 nm.

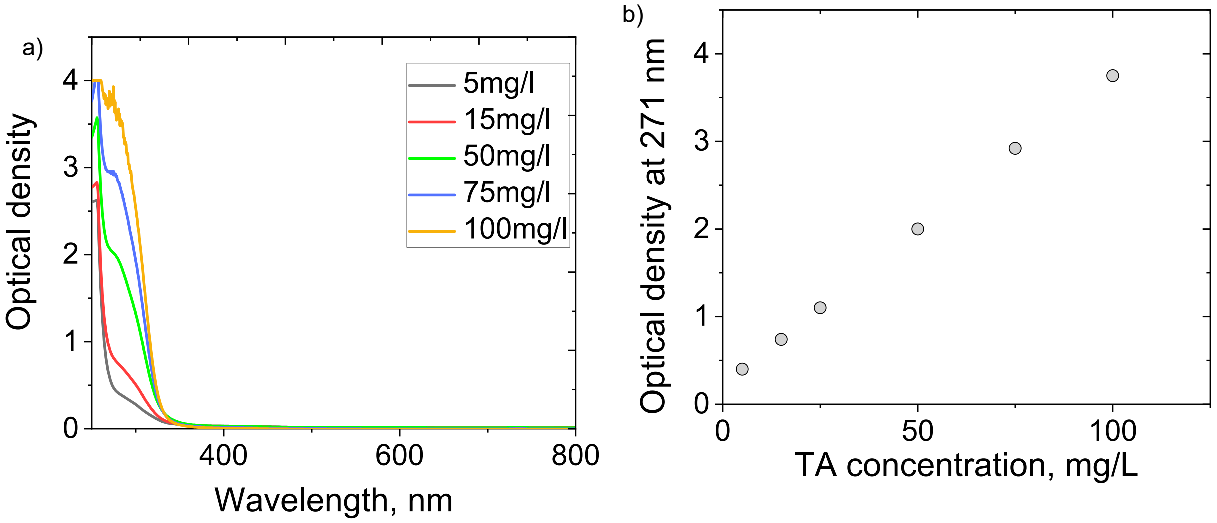

Figure S7. a) UV-VIS spectra of TA aqueous solutions of different concentrations; b) dependence of the optical density at 271 nm (peak of TA absorption) on TA concentration.

The concentration of TA absorbed in the gels can be estimated as follows. The optical density at 271 nm is 1.8 for the gel PEGMA11/PEGDA5/f-PVA2/TA, which was cut into pieces by ultrasound and dispersed in water at a concentration of 0.5 wt%. According to the calibration curve (Figure S5b), TA concentration in this dispersion is 45 mg/L. Accordingly, TA concentration in

the initial gel is (45 mg/L) / 0.005 = 9 g/L = 0.9 wt%. This means that the TA concentration in the gel is close to the TA concentration of the outer solution in which the gel was immersed (1 wt%).

7. LEACHING OF TA FROM THE GELS

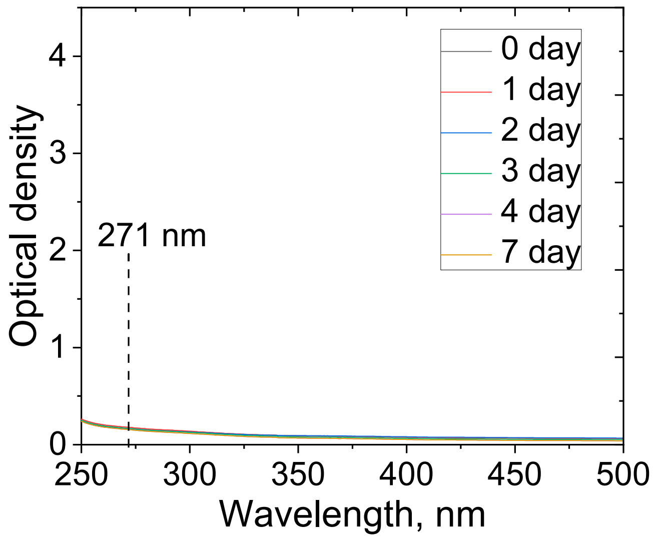

Figure S8. UV-VIS spectrum of PBS, measured after immersion of PEGMA11/PEGDA5/f-PVA3/TA hydrogel for several days. The dashed line indicates the position of the peak of TA at 271 nm.

8. ADDITIONAL SWELLING DATA

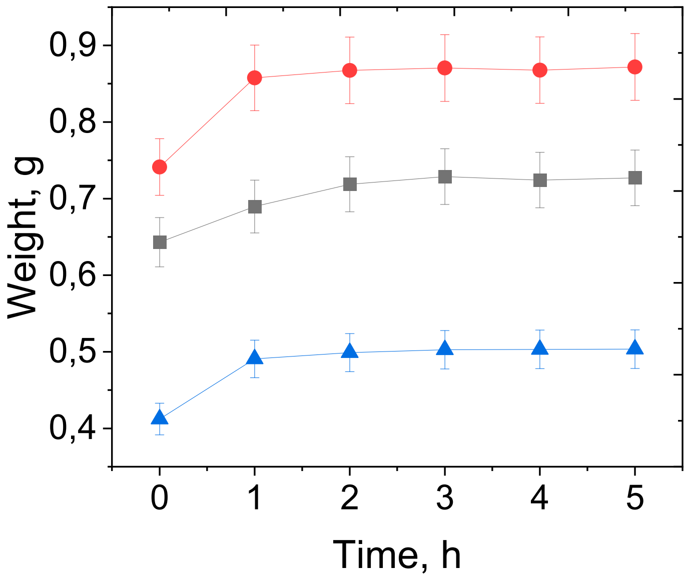

Figure S9. Mass vs. time *of the PEGMA11/PEGDA5 hydrogel (red); PEGMA11/PEGDA5/f-PVA3 hydrogel (black); and PEGMA11/PEGDA5/f-PVA3/TA hydrogel (blue), immersed into the 0.9 wt% aqueous solution of NaCl.*

Table S2. Photos of the prepared gels before and after swelling. Corresponding diameters of the gels are indicated in the photos.

Hydrogel	before swelling	after swelling
PEGDA11/PEGMA5	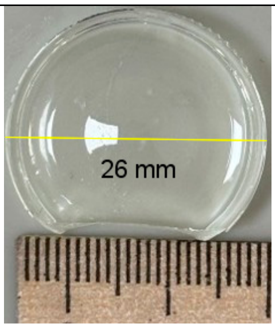	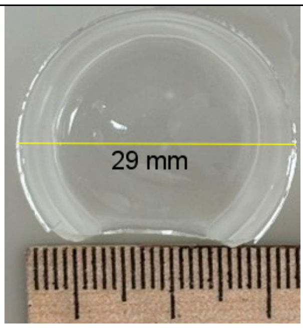
PEGDA11/PEGMA5/f-PVA3	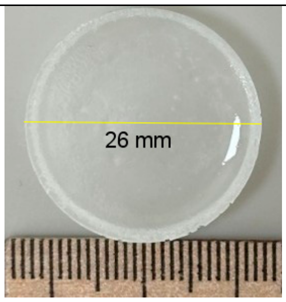	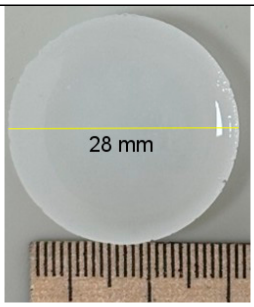

PEGDA11/PEGMA5/f-PVA3/TA	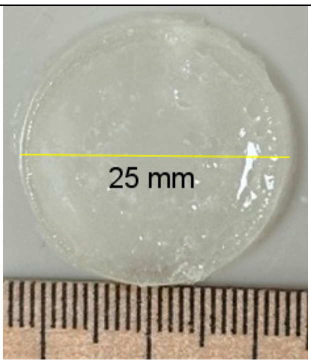 	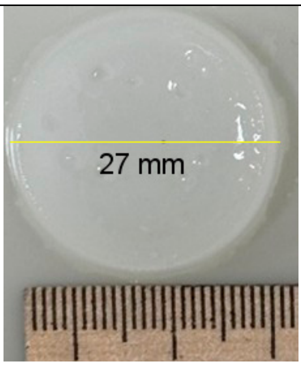

9. ADDITIONAL BIOCOMPATIBILITY DATA

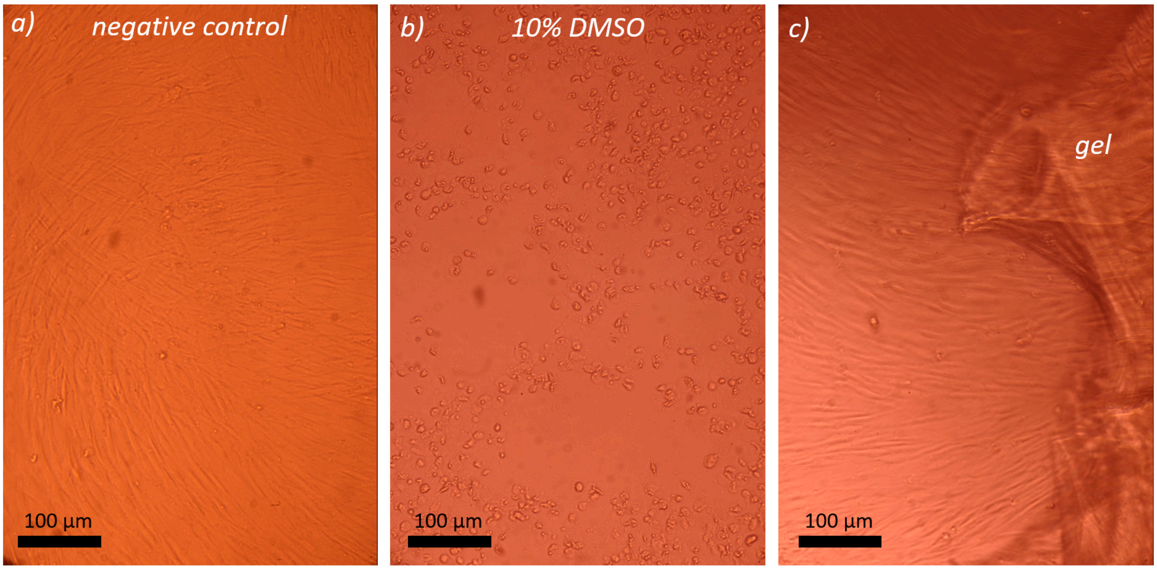

Figure S10. Optical microscopy pictures of fibroblasts in the negative control experiment (a); positive control, e.g., contact of cells with 10% DMSO (b); and in direct contact with the PEGMA11/PEGDA5/f-PVA3/TA hydrogel (c).

10. ESTIMATION OF THE EXPERIMENTAL ERRORS

The standard deviations **SD** of the obtained values of the elastic modulus, cell viabilities, and swelling coefficients were calculated by the addition of the statistical errors σ_{st}, estimated from two to four independent measurements of the reproduced samples and systematic errors σ_{system} of each experimental method:

$$SD = \sqrt{\sigma_{st}^2 + \sigma_{system}^2} .$$

The corresponding statistical error of N independent measurements x_i with mean value $\overline{x} = \frac{1}{N}\sum_{i=1}^{N} x_i$ was obtained from the following formula:

$$\sigma_{st} = \sqrt{\frac{1}{N(N-1)}\sum_{i=1}^{N}(x_i - \overline{x})^2} .$$

The relative systematic error $\varepsilon_{\text{system}}$ of the rheological measurements, swelling behavior, and cell viability were estimated as 10, 5, and 20%, respectively. The absolute systematic error σ_{system} for each mean value $\overline{x}$ was calculated as: $\sigma_{system} = \varepsilon_{\text{system}} \cdot \overline{x}$.
